# Neurocognitive Processing of Facial Emotion Recognition in Individuals With Depression and Suicidal Ideation: An Eye-Tracking and EEG Study

**DOI:** 10.31083/AP44992

**Published:** 2026-02-25

**Authors:** Qianlan Yin, Huijing Xu, Ying Zhu, Meng Liang, Qian Jiang, Bin Zhao, Taosheng Liu

**Affiliations:** ^1^Department of Psychiatry, Faculty of Psychology and Mental Health, Naval Medical University, 200000 Shanghai, China; ^2^Department of Radiology, Shanghai Changzheng Hospital, Naval Medical University, 200000 Shanghai, China; ^3^The 904th Hospital of Joint Logistics Support Force, The Chinese People’s Liberation Army (PLA) Mental Health Center, 213003 Changzhou, Jiangsu, China; ^4^Department of Medical Psychology, Chinese People’s Liberation Army Navy No. 905 Hospital, 200052 Shanghai, China

**Keywords:** suicide ideation, multimodal approach, eye-tracking, electroencephalography

## Abstract

**Background:**

Suicide ideation (SI) is a critical concern, and understanding its neurocognitive underpinnings is essential for improved risk assessment. This study investigates altered neurocognitive processing during face recognition in individuals with depression and SI, utilizing a multimodal approach combining eye-tracking, electroencephalography (EEG), and deconvolution modeling.

**Methods:**

Eye-tracking and EEG data were recorded during face recognition tasks in individuals with depression, with and without SI. We analyzed visual attention patterns (fixation durations, saccadic velocities) and event-related potentials to emotional stimuli. Deconvolution analysis separated microsaccade-related activities (like regression-based event-related potential (rERP) and regression-based fixation-related potential (rFRP)).

**Results:**

Individuals with SI exhibited attentional biases toward emotional faces, characterized by shorter first fixation durations and faster saccadic velocities. Reduced rERP amplitudes in response to surprise and decreased rFRP amplitudes during sad conditions were also observed, suggesting altered neural responses. Integrating eye-tracking and EEG data (the area under the curve (AUC) = 0.771) improved the accuracy of detecting SI compared to eye-tracking alone (AUC = 0.643).

**Conclusions:**

These findings provide novel evidence for altered neurocognitive processing of emotional faces in individuals with depression and SI. The multimodal approach highlights the potential of combining eye-tracking and EEG measures as biomarkers for identifying individuals at risk. Future research should focus on larger, more diverse samples and longitudinal designs to validate these findings and translate them into clinical applications.

## Main Points

1. The study utilized a multimodal approach combining eye-tracking, electroencephalography (EEG), and deconvolution modeling to successfully analyze and reveal behavioral and electrophysiological differences in visual attention and emotional processing in individuals with suicide ideation (SI).

2. Individuals with depression and SI exhibit distinct neurocognitive patterns during facial emotion recognition, characterized by attentional biases towards emotional faces (e.g., shorter first fixation durations, faster saccadic velocities) and altered neural responses to surprise and sadness (reduced regression-based event-related potential (rERP) and regression-based fixation-related potential (rFRP) amplitudes).

3. Integrating eye-tracking and EEG data significantly enhanced the accuracy of detecting SI (area under the curve, (AUC) = 0.771) compared to using eye-tracking data alone (AUC = 0.643), highlighting the potential of multimodal biomarkers for identifying at-risk individuals.

## 1. Introduction

Suicide represents a major global health concern, impacting individuals, families, and communities across all socioeconomic strata and geographical locations; the ability to prevent suicide through personalized interventions highlights the necessity for more objective and reliable diagnostic methodologies to supplement interview-based risk assessments [1, 2]. The subjective nature of clinical interviews and questionnaires can lead to misinterpretations due to semantic nuances and the challenges some patients face in articulating their thoughts and emotions [3, 4]. This necessitates the exploration of complementary methods that can offer a more objective assessment of suicide risk, potentially circumventing the limitations inherent in self-reporting and clinician interpretation [5].

Recent studies suggest that depression and suicide ideation (SI) can influence emotional perception. Individuals with major depressive disorder and SI exhibit distinct patterns in processing emotional faces, with impaired processing of information from the eye region and difficulty in attention towards emotional information [6, 7, 8]. This could manifest as difficulty in identifying facial expressions or an altered allocation of attentional resources when viewing faces [9]. This alteration is particularly evident in the recognition of negative emotional expressions, where individuals with depression often exhibit a bias towards interpreting neutral expressions as negative and show reduced attentional engagement with positive stimuli [10, 11, 12]. This phenomenon may stem from a dysfunctional processing of emotional information, often manifesting as an attentional bias towards negative emotional cues [13]. This attentional bias, particularly in the context of suicide-related information, has been identified as a potential cognitive marker for suicide risk [14]. Individuals with depression and SI is associated with notable impairments in facial recognition, particularly in distinguishing emotional from neutral expressions [15, 16]. While studies on depression generally indicate a reduced ability to recognize neutral emotions compared to pleasant or sad ones, research on individuals with depression and SI presents mixed findings regarding specific emotional facial recognition deficits. For instance, one study found that individuals with a history of suicide attempts made more errors identifying disgust than controls [17]. Another indicated abnormal brain activity in response to angry faces, with increased activity in the right orbitofrontal cortex and decreased activity in the right superior frontal gyrus [18]. Conversely, some research suggests that individuals with depression and SI may have improved accuracy in recognizing fearful expressions compared to those without SI, with regression analyses indicating difficulties in recognizing neutral facial expressions among those at high risk for suicide [19]. Therefore, capturing the neurophysiological responses associated with this attentional bias in visual processing may provide a more nuanced understanding of the cognitive and emotional mechanisms underlying suicide risk in individuals with depression.

Previous research has extensively utilized neurophysiological techniques like electroencephalogram (EEG) and eye-tracking to investigate emotional processing and attention. EEG provides a non-invasive measure of brain electrical activity, allowing for the examination of event-related potentials that are time-locked to specific events or stimuli, such as the presentation of faces [20, 21]. EEG studies have frequently demonstrated altered event-related potentials in individuals with depression when processing emotional stimuli, often indicating atypical attentional allocation and emotional regulation [22, 23]. Specifically, studies have shown attenuated P300 amplitudes to positive stimuli and enhanced late positive potentials to negative stimuli in individuals with depression, reflecting altered emotional salience processing [24]. Similarly, eye-tracking offers precise measurements of eye movements, such as microsaccades, which are involuntary, small, and rapid eye movements that occur during visual fixation; these movements are closely linked to attention and cognitive processing [25]. Eye-tracking studies have revealed atypical gaze patterns, such as reduced scanning of the eye region in emotional faces and prolonged fixation on negative stimuli, reflecting an underlying attentional bias towards threat-related cues [26]. Particularly, eye-trackers and EEG are powerful tools to investigate these neural mechanisms in the context of SI [7, 27, 28, 29]. For instance, research indicates that individuals with SI exhibit sustained eye-tracking biases towards suicide-relevant information, alongside distinct patterns of brain activation in frontal and temporal regions [5, 14]. EEG studies in individuals with SI have also shown altered P300 and late positive potential responses to suicide-related words and images, indicating aberrant emotional and cognitive processing [22]. While these individual methodologies have provided valuable insights into the neural mechanisms underlying face recognition and emotional processing, they often face limitations. Traditional EEG analysis can struggle with overlapping neural signals, especially in the presence of continuous eye movements, making it challenging to precisely attribute brain activity to specific cognitive events or subtle oculomotor behaviors. Similarly, eye-tracking alone, while offering precise measurements of overt attention, cannot directly capture the underlying neural correlations of these processes. This limitation underscores the necessity for an integrated approach that can simultaneously capture both overt behavioral responses and their underlying neurophysiological signatures.

This study introduces a significant methodological advancement by combining eye-tracking, EEG, and linear deconvolution modeling. This integrated approach offers unique value in overcoming the limitations of previous studies, as eye-tracking provides precise measurements of eye movements, such as microsaccades—involuntary, small, and rapid eye movements linked to attention and cognitive processing. Simultaneously, EEG captures brain electrical activity, allowing for the examination of event-related potentials. Crucially, the application of deconvolution modeling enables the disentanglement of overlapping EEG signals, allowing for the precise isolation and characterization of both stimulus-related potentials and microsaccade-related potentials [30]. This sophisticated analytical technique statistically differentiates brain responses elicited by multiple, potentially overlapping event types, providing a more accurate understanding of attention, emotion processing, and cognitive control by using the timing differences between eye movements and accounting for unrelated factors [30, 31]. Hence, examining microsaccade-related potentials during face recognition can provide valuable insights into the neural activity associated with attention, emotion processing, and cognitive control in individuals with depression and SI [32, 33]. Specifically, this combined methodology allows for a comprehensive investigation into how subtle eye movements, such as microsaccades, are modulated during face recognition in individuals with SI, offering a more sensitive and objective way to detect suicide thoughts [7, 14, 34]. By precisely capturing the interaction between eye movements and brain responses, this method improves upon single-modality or less advanced techniques, offering deeper insights into the cognitive and neural mechanisms behind suicide risk in individuals with depression.

Building upon prior research highlighting the significance of emotion regulation and cognitive control in understanding suicide behavior, this study employs a novel combination of electroencephalography, eye-tracking, and deconvolution modeling to investigate the neural and cognitive processes involved in face recognition among individuals with depression and SI. By examining microsaccade-related potential, our aim is to elucidate the interplay between attention, emotion processing, and cognitive control in this population, thereby bridging the gap between underlying neural mechanisms and the clinical manifestations of suicide risk in depression.

## 2. Methods

### 2.1 Participants

A cohort of individuals diagnosed with depression, with a subset experiencing SI, were recruited for the study. The inclusion criteria included meeting the diagnostic criteria for major depressive disorder based on the Diagnostic and Statistical Manual of Mental Disorders (Version 5). Exclusion criteria included a history of neurological disorders, severe head trauma, or current substance abuse, as these conditions could confound the EEG and eye-tracking data. The study received approval from the Ethics Committee of the 904th Hospital of Joint Logistics Support Force (approval number SFYEC-202403-C9), and all participants provided informed consent in accordance with established ethical guidelines. A priori power analysis (G*Power, version 3.1.9.7, Heinrich-Heine-Universität Düsseldorf, Düsseldorf, Germany; https://www.gpower.hhu.de) for a repeated-measures ANOVA with two groups and seven measurements indicated that 26 participants were required to detect a medium effect size (*f* = 0.25) with α = 0.05 and 95% power. Eventually, a total of 59 participants (35 with depression and SI, 24 with depression but no SI) were included in the final analysis, exceeding the required sample size and ensuring adequate statistical power.

### 2.2 Clinical and Behavioral Assessments

The severity of depression was assessed using the Beck Depression Inventory-II (BDI-II) alongside a structured clinical interview [35]. The BDI-II is a widely used self-report instrument designed to measure the severity of depressive symptoms. Participants rate the intensity of their symptoms experienced over the preceding week, with elevated scores indicative of greater depressive symptomology. A score exceeding 14 is often interpreted as indicative of depression [36]. SI was evaluated using the Beck Scale for Suicide Ideation (BSI), which assesses the intensity and frequency of suicide thoughts [37]. In BSI, the presence of SI is determined by a positive response (score of 1 or 2) on BSI item 4 (“Thoughts of suicide”) or item 5 (“Suicide acts/attempts”), provides a more precise and clinically focused definition for the SI group [38, 39, 40]. Using these specific items, which directly assess the existence of suicide thoughts or plans/attempts, can indeed lead to a more homogeneous and accurately defined the SI group compared to using the total BSI score, especially if the total score could be inflated by items less directly related to active ideation. This categorization, along with clinical interviews conducted by experienced mental health professionals, guided the division of participants into different risk categories in our research. At baseline, demographic characteristics including age, gender, and education level were assessed for both groups. While formal matching procedures were not employed for these demographic variables, statistical comparisons confirmed their comparability between the SI and NSI (no SI) groups. It is important to note that, as anticipated, depression severity (as measured by the BDI-II) significantly differed between the two groups; therefore, BDI-II scores were considered as a covariate in all relevant statistical analyses to control for this potential confound.

### 2.3 Stimuli and Procedure

**Fig. 1.**
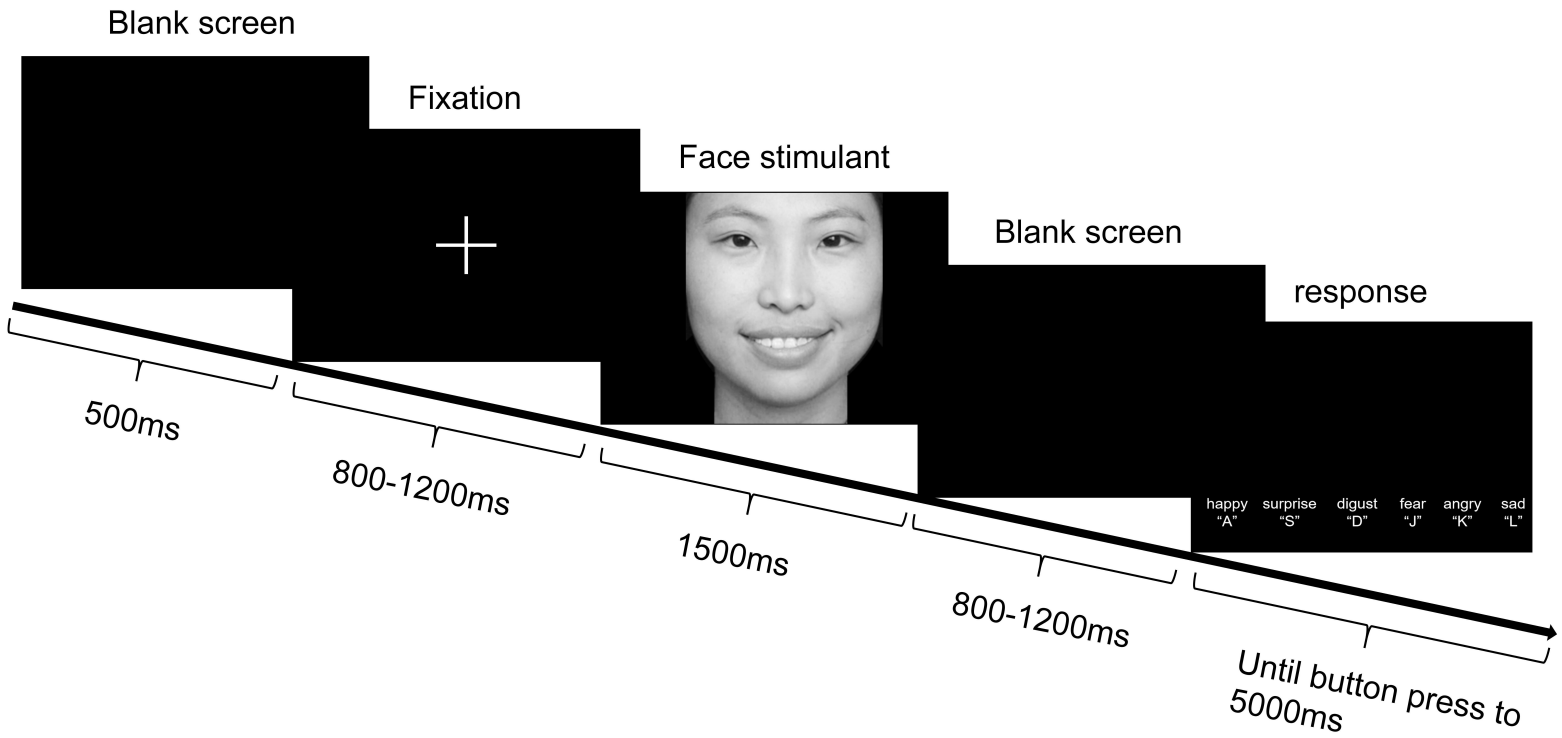
**A paradigm of experimental procedure for emotional face recognition task**. Note: Each trial proceeded as follows: First, a white fixation point appeared in the center of the screen for 500 ms. Subsequently, a blank screen was displayed for a randomized duration of 800 to 1200 ms. Next, an emotional face stimulus was presented for 1500 ms. After the stimulus disappeared, another blank screen appeared for a randomized duration of 800 to 1200 ms. Finally, the emotional face selection interface was displayed. In the selection interface, participants pressed the selection key to proceed to the next trial. If no response was made, the selection interface automatically advanced to the next trial after 5000 ms.

Participants were presented with a series of facial stimuli displaying a range of emotional expressions (e.g., happiness, sadness, anger, fear, surprise, disgust and neutral). This emotional face recognition task utilized the localized Chinese Affective Picture System, compiled by Bai *et al*. [41]. This database has been validated for use in patients with bipolar disorder, schizophrenia, depression, and other conditions, ensuring its suitability for our target population. Based on the data provided by the Chinese Affective Picture System (CAPS), images with obvious hair or beard occlusion were excluded, and gender factors were balanced. Twenty face images were selected for each of the six basic emotions, and twenty face images for the neutral emotion, resulting in a total of 140 face images used as experimental stimuli. This selection process ensured a comprehensive representation of emotional expressions relevant to the study of depression and SI. To further standardize the stimuli, obvious moles, acne scars, and blemishes in the selected images were blurred using Adobe Photoshop, and all images were adjusted to the same brightness. The resolution of all images was adjusted to 260 × 300 pixels. Participants were required to identify the category of emotional face images and press the corresponding key, ensuring their fingers remained poised above the designated keys. The screen provided a visual guide, indicating the corresponding direction for each key, thereby eliminating the need for participants to visually locate the keys on the keyboard. This setup aimed to streamline the response process and minimize potential distractions, allowing for a more focused and efficient task performance. The experiment comprised 280 formal trials, with a break scheduled after every 140 trials and each stimulus presented 2 times. Stimuli presentation duration was controlled and consistent across participants, with each image presented for 1500 ms, with fixed 2-s intervals between image presentations (see Fig. 1). The order of stimuli presentation was randomized to minimize order effects. Before the formal task began, participants completed practice trials until they demonstrated proficiency with the keyboard operations. A detailed paradigm of the experiment is illustrated in Fig. 1. E-Prime 2.0.8.22 (Psychology Software Tools, Inc., Sharpsburg, PA, USA, https://www.pstnet.com) was used for presentation and to record participants’ reaction times and accuracy rates.

Participants were instructed to maintain fixation on a central cross while viewing the faces, ensuring consistent visual input across all trials. Eye-tracking data were collected using the Tobii TX300 desktop eye tracker (Tobii AB, Danderyd, Sweden, https://www.tobiipro.com) from Sweden, with a sampling rate of 300 Hz. The display used for presenting task stimuli was its accompanying Tobii TX Display, with a resolution of 1920 × 1080 pixels and a refresh rate of 120 Hz. During the experiment, participants were required to sit 60 cm away from the screen. Before the experiment, the eye tracker’s built-in 9-point calibration mode was used for calibration. After the accuracy reached the standard, the formal experiment was conducted.

The EEG data were recorded using a Biosemi 64-channel EEG acquisition system, with electrodes positioned according to the international 10–20 system. During acquisition, the system’s built-in “CMS” and “DRL” electrodes served as reference electrodes, while offline analysis employed the whole-brain averaging as a reference. EEG recording commenced once the contact impedance between the electrodes and the scalp was below 10 kΩ, ensuring high signal quality. The filter bandpass was set to 0.01–1000 Hz, with AC sampling at a rate of 1000 Hz, optimized for capturing both slow cortical potentials and faster transient activity.

### 2.4 Data Analysis

Descriptive statistical analysis was performed on demographic data, clinical assessment scale scores, and task behavioral data, including reaction times and accuracy rates. For continuous variables, normality of distribution was assessed using the Shapiro-Wilk test. Variables that were normally distributed are presented as mean ± standard deviation (SD), while those that were not normally distributed are reported as median (interquartile range [IQR]). Independent sample *t*-tests or chi-square tests were used to compare the demographic and clinical characteristics of the two groups. Repeated measures ANOVA, with depression scores as a covariate, was used to analyze behavioral data like reaction time and accuracy and eye movements. Statistical significance was set at *p* < 0.05.

Eye-tracking data were preprocessed to detect saccades and fixations using EYE-EEG toolbox (version 1.0, available at https://github.com/olafdimigen/eye-eeg/releases/tag/v1.0, developed by the Donders Institute for Brain, Cognition and Behaviour, Radboud University, Nijmegen, The Netherlands) [42]. Different parameters related to fixations and saccades were extracted, including the number of fixations or saccades within a specific time frame, fixation duration, and saccade amplitude. EEG data were down-sampled to 250 Hz and band-pass filtered to 0.1–40 Hz [43]. Bad channels and bad trials were automarked according to The Harvard Automated Processing Pipeline for Electroencephalography (HAPPE) pipeline and were then visually inspected and removed; then the data was re-referenced to the average of all channels [43].

Using the EYE-EEG toolbox, eye-tracking and EEG data were then synchronized based on shared trigger pulses sent frequently to both systems. The average synchronization error (misalignment of shared trigger pulses after synchronization) was less than 1 ms. Epochs were created from –200 ms to 1000 ms relative to stimulus onset. In the initial step, clean trials were identified based on the absence of missing eye-tracking data, EEG data, or artifacts from –200 ms to 1000 ms relative to stimulus onset. Three criteria were applied: First, trials including either eye blink or gaze measurements outside of the stimulus image were rejected. Second, trials were excluded if the mean gaze position during the 200 ms pre-stimulus interval was not within a quadratic bounding box centered on the fixation cross. Finally, trials containing remaining non-ocular EEG artifacts (defined as voltages exceeding ±120 µV relative to baseline at each channel, after ocular artifact correction) were discarded. In the remaining clean trials, saccade and fixation events were detected using the binocular version of the velocity-based microsaccade detection algorithm by Engbert and Kliegl, as implemented in the EYE-EEG toolbox (velocity threshold: 5 median-based SDs, minimum saccade duration: 8 ms, binocular overlap required). Microsaccades were defined by a minimum amplitude of 0.5° and a peak velocity of 30°/s [44, 45, 46].

Prior to deconvolution analysis, epochs from –200 ms to 1000 ms relative to stimulus onset were created using the remaining clean trials. Then deconvolution analysis was conducted using the procedure outlined by SpieringEhinger and Dimigen [30]. This procedure fits a regression model to the continuous EEG data that estimates Event-Related Potentials(ERPs) time-locked to multiple, potentially overlapping event types [47]. Stimulus- and fixation-related brain responses were modeled and statistically separated using the unfold toolbox (version 1.2, https://github.com/unfoldtoolbox/unfold). For details, the reader is referred to recent tutorial papers explaining this approach in detail [30, 48]. Also, the specific process of our research can also be found in the Supporting Information. Compared with traditional ERP averaging, linear deconvolution modeling has two crucial advantages for analyzing experiments with eye movements [30, 49]. First, the temporal variability between different oculomotor events is leveraged to statistically differentiate the overlapping brain responses elicited by each event type. Second, the model enables statistical control over various nuisance variables known to influence brain responses related to eye movements. Given that these waveforms are estimated within a regression framework, rather than through traditional averaging, they are often termed regression ERPs and regression FRPs (rERPs and rFRP).

For the statistical approach, we first employed a data-driven method to analyze the rERP and rFRP components within a broad time window and across all channels. This approach allowed us to objectively identify the temporal components that exhibited the most pronounced differences between the two groups, minimizing any potential bias in our analysis. To compare the spatial distribution of rERPs and rFRPs between the SI and NSI groups, we conducted a series of *t*-tests at each electrode and time point. To mitigate the risk of Type I errors associated with multiple comparisons, cluster permutation tests (implemented in the *ept_TFCE* toolbox: https://github.com/Mensen/ept_TFCE-matlab). These tests used a cluster-forming threshold of *p* < 0.05 and a cluster alpha level of 0.05 to correct for multiple comparisons, thereby ensuring the robustness of our findings.

To predict SI, all characteristics revealed from the clinical characteristics, eye movement and EEG data were used as features and the suicide ideation as a target variable. Initially, a Random Forest model was trained on the full dataset with the target variable to evaluate feature importance using the random Forest package. The importance of each feature was quantified using the Mean Decrease in Accuracy, and the top features were ranked accordingly. To further assess the predictive power of individual features, we computed the Area Under the Curve (AUC) for each feature by fitting a univariate Receiver Operating Characteristic (ROC) curve using the pROC package (version 1.18.0; https://cran.r-project.org/package=pROC) in R v4.3.2 (R Core Team, Institute for Statistics and Mathematics, Vienna, Austria). Features with an AUC greater than 0.6 were selected for model training to ensure only those with reasonable discriminatory power were retained. These features, along with the group label, were combined into a new dataset for model development. The data were then randomly split into training (80%) and testing (20%) subsets. A Random Forest model with 500 trees was trained on the selected features from the training set. Probabilities for the positive class were obtained from the testing set predictions. The classification performance was evaluated by calculating the AUC of the ROC curve and predicted class labels were generated using a probability threshold of 0.5.

## 3. Results

### 3.1 Demographic and Behavioral Results

Demographic characteristics were comparable between the NSI and the SI groups in our sample. However, as anticipated, the depression scores were significantly different between the two groups (see Table 1). The severity of depression was included as a covariate in all subsequent analyses.

**Table 1.** **Demographic characteristics of participants**.

		Overall	NSI group	SI group	*p*-value
Number		59	24	35	
Gender					0.624
	Male	53 (89.83%)	21 (87.50%)	32 (91.43%)	
	Female	6 (10.17%)	3 (12.50%)	3 (8.57%)	
Education					0.105
	High school	20 (33.90%)	4 (16.67%)	16 (45.71%)	
	Junior college	25 (42.37%)	14 (58.33%)	11 (31.43%)	
	Senior College	11 (18.64%)	5 (20.83%)	6 (17.14%)	
	Master or above	3 (5.09%)	1 (4.17%)	2 (5.71%)	
Marital status					0.951
	Single	44 (74.58%)	18 (75.00%)	26 (74.29%)	
	Married	13 (22.03%)	5 (20.83%)	8 (22.86%)	
	Divorce	2 (3.39%)	1 (4.17%)	1 (2.86%)	
Age		24.00 (22.00–30.00)	25.00 (22.75–31.25)	23.00 (21.50–28.50)	0.462
Depression		26.14 ± 14.26	16.96 ± 13.08	32.43 ± 11.44	<0.001
Suicide (Beck)		2.00 (0.00–3.00)	0.00 (0.00–0.00)	3.00 (2.00–4.00)	<0.001

Note: NSI, Non-suicide ideation; SI, suicide ideation.

Although no significant differences emerged between the NSI and the SI groups in the behavioral measures of reaction time and accuracy, the overarching between-group effect did not reach statistical significance. However, the influence of emotional conditions on these behavioral outcomes was indeed significant (Reaction Time(RT): F = 11.67, *p* < 0.001, Df = 6, η^2^ = 0.160; Accuracy(ACC): F = 45.62, *p* < 0.001, Df = 6, η^2^ = 0.430).

### 3.2 Eye Movements Results

#### 3.2.1 Eye Region Concentration and Shorter First Fixation Durations in the SI Group

**Fig. 2.**
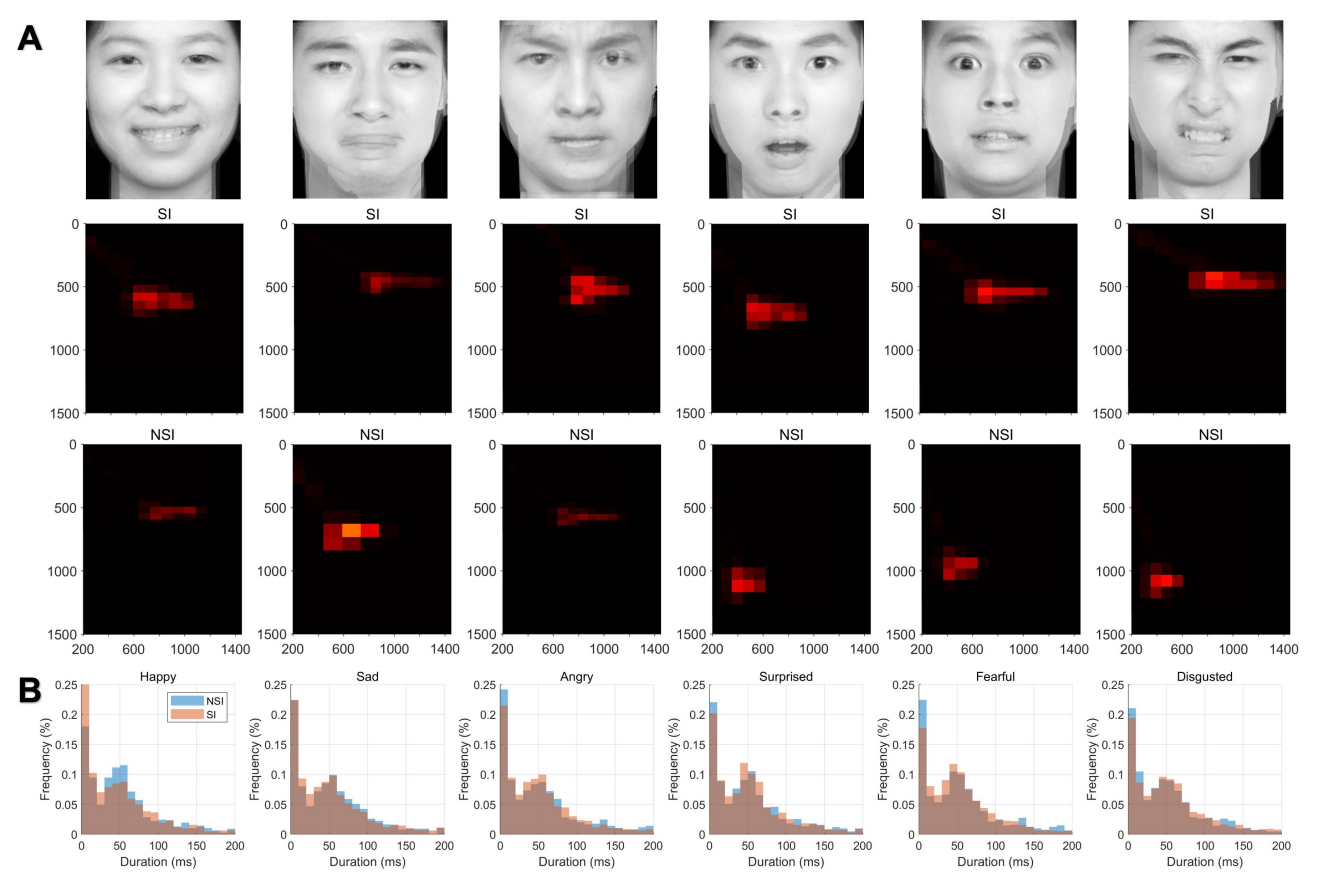
**Fixation density plots and fixation duration distributions for SI and NSI group**. (A) Heatmaps depicting fixation locations for the groups across six emotional stimuli. The intensity of the heatmaps indicates fixation density on facial regions. (B) Histograms showing the distribution of fixation durations for the groups across the same six emotional stimuli. The x-axis represents fixation duration (ms), and the y-axis represents the percentage of fixations.

The distribution of fixations on various Areas of Interest (AOIs) of emotional faces was analyzed to investigate group differences in attentional allocation during face recognition. The interactive effects of group and AOI on fixation counts were substantial (F = 19.39, *p* < 0.001, Df = 5, η^2^ = 0.040), while the effects of emotions were not significant. The SI group exhibited a distribution of fixations more concentrated on the eyes (t = 3.802, *p* < 0.001, see Fig. 2A), but the NSI group showed a more dispersed pattern. Moreover, although the duration was not significant, which also shows in Fig. 2B, the first fixation duration was shorter in the SI group than that in the NSI group (F = 54.58, Df = 1, *p* < 0.001, η^2^ = 0.004).

#### 3.2.2 Faster Saccadic Velocities in SI Group

**Fig. 3.**
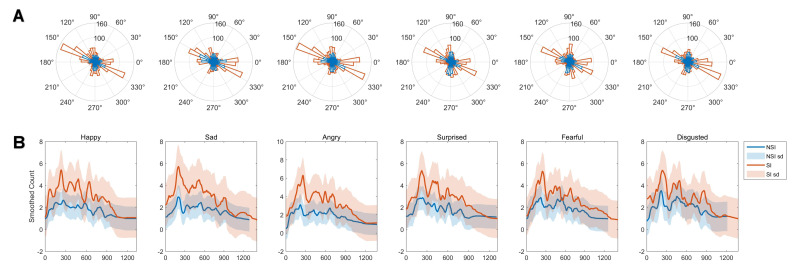
**Distribution of saccade directions and saccade rates following six emotional stimuli**. (A) Polar plots illustrating the distribution of saccade directions for each emotional stimulus. The radial axes represent the frequency of saccades at corresponding angles. (B) Line graphs showing the rate of saccades over time for each emotional stimulus. Shaded areas represent ±1 standard error of the mean.

The SI group exhibited significantly faster saccadic velocities compared to the NSI group, across all emotional conditions (F = 25.78, *p* < 0.001, Df = 1, η^2^ = 0.014, see in Fig. 3A). This significant main effect of group on saccade velocity suggests potential neurophysiological differences between the two clinical populations. However, the saccade count did not show significant differences between the two groups (F = 0.432, *p* = 0.514), as presented in Fig. 3B.

### 3.3 EEG Results

#### 3.3.1 Decreased rERP Amplitudes to Surprise in SI Group

**Fig. 4.**
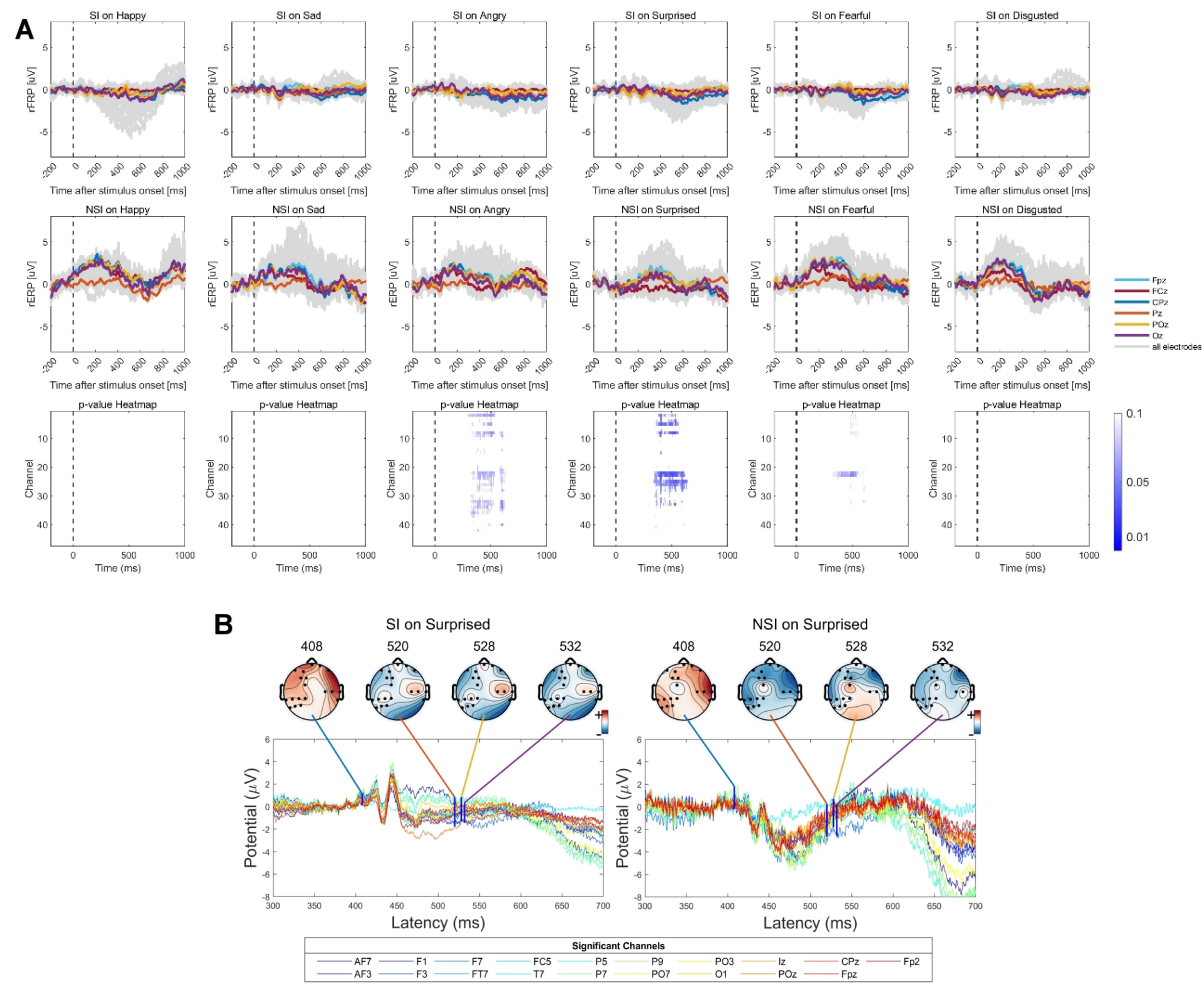
**Grand-average overlap-corrected regression-based Event-Related Potential (rERP) waveforms and group-level differences under six emotional conditions**. (A) Grand-average rERP waveforms for all channels are displayed in dark-grey lines, with electrodes at central positions highlighted in colors. The lower panel shows results of the Threshold-Free Cluster Enhancement (TFCE)-based *t*-test on interval 0–1000 ms across all channels, indicated by blue dotted lines representing statistical significance. The colored curves indicate channels with significant group-level differences in rERP amplitude after the presentation of surprised faces. (B) Topographic maps of significant group-level differences in rERP between SI and NSI groups evoked by surprised faces, shown at specific channels and latency time points. The time frames correspond to the minimum *p*-values identified in panel A (marked by bold blue vertical lines).

Fig. 4A depicts the rERPs elicited by the stimulus onset as a function of emotional condition. Under different emotional states, the amplitude range of the central electrodes was illustrated while the statistical results across all channels and timeframe were presented in the bottom of Fig. 4A. These findings highlight the significance of regression ERPs in capturing subtle yet meaningful variations in neural processing during emotional face recognition. As shown in Fig. 4A, the SI group exhibited significantly different wave amplitudes after 450 ms when exposed to emotional facial stimuli, particularly under the surprise condition, compared to the NSI group. This divergence suggests altered neural processing of emotional stimuli in individuals with SI. Further, Fig. 4B, which focuses on the differences in rERPs under the surprise condition, reveals that the SI group had different amplitude with the NSI group from 368–628 ms. The most statistically significant *p*-value presented at channel P7 (timeframe = 408, *p* = 0.026) and FT7 (timeframe = 520/528/532, *p* = 0.027/0.028/0.029), both located near the left lateral area. The SI group shows significantly lower amplitude in identifying surprise at these time frames compared to the NSI group, suggesting that the SI group are less sensitive to surprise emotion than the NSI group.

#### 3.3.2 Reduced rFRP Amplitudes to Sad Faces in SI Group

**Fig. 5.**
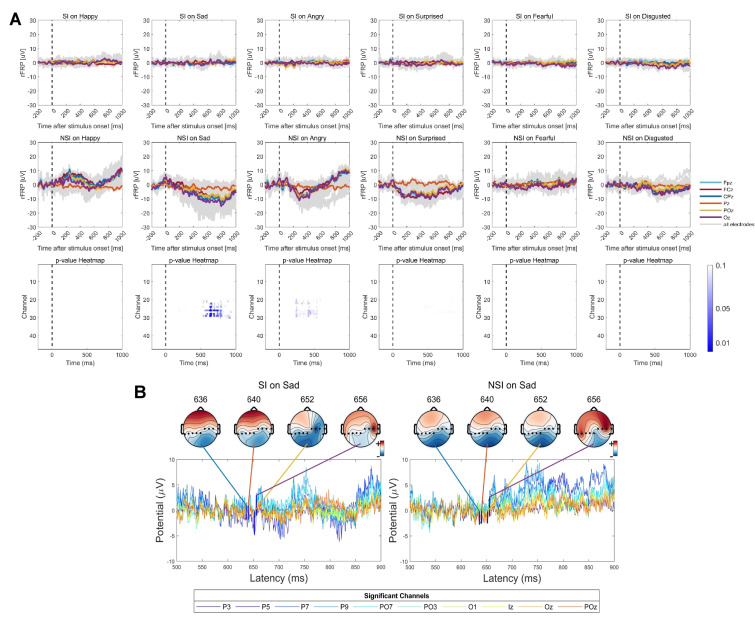
**Grand-average overlap-corrected regression-based Fixation-Related Potential (rFRP) waveforms and group-level differences under six emotional conditions**. (A) Grand-average rFRP waveforms across all channels are depicted in dark-grey lines, with central electrodes highlighted in colors. The lower panel shows TFCE-based *t*-test results over the 0–1000 ms interval across all channels, with significant clusters indicated by blue dotted lines. Colored curves represent channels showing significant group-level differences in rFRP amplitude after sad face stimuli. (B) Topographic maps illustrating significant group-level differences in rFRP between SI and NSI groups evoked by sad faces at specific channels and latency time points. Time frames correspond to minimum *p*-values shown in panel A (indicated by bold blue vertical lines).

Fig. 5A depicts the saccade-related potentials as a function of emotion condition, time, and group, time-locked to saccade onsets. When comparing saccade-related potentials during sad conditions between the SI group and the NSI group, significant differences emerged within the 564–920 ms time window after microsaccade onset. Further, Fig. 5B illustrated the significant group-level differences in rFRP after sad faces presented at specific channels and time frames. The observed significant differences at electrode PO3 during the 636–656 ms time frames, with the SI group exhibiting lower amplitude compared to the NSI group, suggest a promising neural substrate that may serve as a potential biomarker to distinguish between individuals with and without SI.

#### 3.3.3 Enhanced Predictive Accuracy With Multimodal Eye-Tracking and EEG Data

**Fig. 6.**
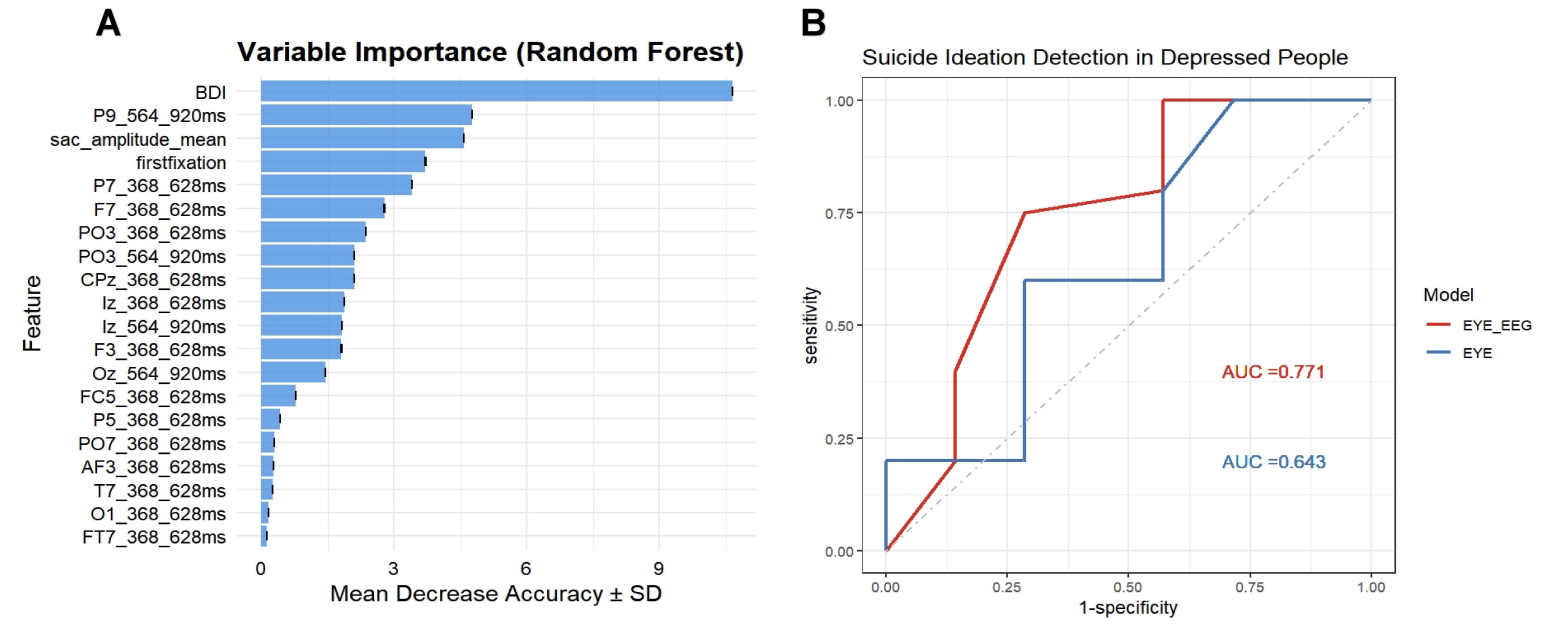
**Multimodal feature contribution and classification performance for suicide ideation detection in individuals with depression**. (A) Variable importance plot derived from the Random Forest model. The Beck Depression Inventory (BDI) score ranks highest in importance, followed by eye movement measures including saccade amplitude (sac_amp), first fixation duration, and EEG features such as rERP and rFRP components at specific time windows (368–628 ms and 564–620 ms) and channels. (B) Receiver Operating Characteristic (ROC) curves comparing classification performance between models using combined eye movement and EEG features (EYE-EEG, Area Under the Curve (AUC) = 0.771) versus eye movement features alone (EYE, AUC = 0.643) for detecting suicide ideation in depressed individuals.

To identify the neuromarker of SI in the depression group, ROC analysis was performed on the features extracted from stimulus related ERPs, microsaccade related potentials, eye-tracking data and clinical scales. First, a random forest was trained on 80% of total data and tested on the rest 20% data. The feature importance was ranked based on how each feature decreases the impurity of the forest. The most important features are selected to perform the ROC analysis, and the results were shown in Fig. 6. The variable importance plot from the Random Forest model (Fig. 6A) highlights the most informative features contributing to the classification. The Beck Depression Inventory score emerged as the most influential variable, followed by EEG features such as rFRP components at 564–920 ms time frame under sad conditions and eye movement metrics including saccade amplitude and first fixation duration. Several EEG-derived features at specific channels and time windows (e.g., F7, P7, PO3) also contributed to model performance. Moreover, the ROC analysis (Fig. 6B) demonstrates that incorporating both eye movement and EEG features slightly improves the model’s ability to detect SI in individuals with depression compared to using eye movement features alone. Specifically, the EYE-EEG model achieved an AUC of 0.771, outperforming the EYE model with an AUC of 0.643. Although both models show modest discriminatory power, these results indicate that integrating multimodal physiological signals may enhance the performance of predictive models. For the EYE-EEG model, the confusion matrix of the data showed: Accuracy = 0.717 (95% CI: 0.577–0.832), Sensitivity = 0.609, and Specificity = 0.800. The model yielded an F1 Score of 0.762 and a Balanced Accuracy of 0.704, highlighting a reasonably balanced ability to detect both positive and negative cases.

## 4. Discussion

The present study aimed to investigate the neurophysiological and oculomotor correlates of face recognition in individuals with depression and SI, utilizing a combination of EEG, eye-tracking, and deconvolution modeling techniques to identify potential biomarkers for SI and to elucidate the underlying cognitive and affective processes associated with emotional face processing in this population. The findings revealed several key differences between the SI group and the NSI group in terms of eye movement behavior, stimulus-related ERPs, and microsaccade-related potentials. Eye-tracking results indicated that the SI group exhibited distinct patterns of visual attention during face recognition, characterized by shorter first fixation durations and faster saccadic velocities when viewing emotional faces, while EEG results showed under surprise and sad condition that the SI group had different amplitude compared to the NSI group, suggesting altered neural processing of emotional stimuli in individuals with SI. Moreover, the ROC analysis demonstrates that incorporating both eye movement and EEG features slightly improves the model’s ability to detect SI in individuals with depression compared to using eye movement features alone. These findings highlight the potential of multimodal physiological signals as valuable tools for detecting SI in individuals with depression.

### 4.1 Attentional Biases in Suicide Ideation: Eye-Tracking Evidence

In the current study, compared to the NSI group, the SI group exhibited distinct patterns of visual attention during face recognition, characterized by shorter first fixation durations and faster saccadic velocities when viewing emotional faces, which is consistent with previous research indicating attentional biases and altered processing of emotional stimuli in individuals with SI. These findings suggest that individuals with SI may exhibit a heightened sensitivity to emotional stimuli, leading to a more rapid and superficial processing of facial expressions [27]. The quicker saccadic velocities shown by the SI group may indicate a more active and vigilant scanning of the visual environment, potentially reflecting an attempt to identify potential threats or sources of emotional distress [14]. This heightened sensitivity and vigilance may be indicative of underlying anxiety or hypervigilance related to perceived dangers or negative emotional states. Moreover, the reduced first fixation duration observed in the SI group may reflect an attempt to avoid or disengage from emotionally salient stimuli, possibly as a coping mechanism to regulate negative emotions or to minimize exposure to emotionally distressing content. Notably, although we observed statistically significantly shorter first fixation duration of the SI group, the corresponding effect size was very small. This indicates that while the finding is unlikely due to chance and reflects a genuine group difference, the magnitude of this effect is limited and explains only a small proportion of the variance. Therefore, the practical or clinical utility of these singular measures as standalone biomarkers is constrained. However, these subtle effects contribute valuable information that in contrast to individuals without SI, those with suicide thoughts exhibit impairments in processing information from the eye region.

### 4.2 Decoding Emotional Processing in Suicide Ideation: Insights From Event-Related Potentials 

Our findings suggest that individuals with SI may exhibit altered sensitivity to surprise and sad emotions. This could reflect attentional bias and emotional processing deficits correspondence with earlier research [27, 50, 51]. The reduced amplitude in response to surprise may suggest a blunted emotional reactivity or a tendency to disengage from emotionally salient stimuli among individuals with SI. The lower amplitude observed in the SI group during the 564–920 ms time frame suggests impaired cognitive processing or reduced attentional resources allocated to the processing of emotional stimuli. In contrast to rERP, which directly measures event-related potentials, rFRP is derived from deconvolution analysis, which separates the pure ERP signal from microsaccade artifacts. This allows rFRP to specifically reflect the neural activity associated with microsaccadic eye movements and provide deeper insights into the cognitive and sensorimotor processes underlying visual attention and perception [30, 52]. In our study, the SI group exhibited smaller rFRP amplitudes compared to the NSI group under sad conditions, suggesting robust differences in the neural mechanisms underlying microsaccade generation and their relationship to emotional processing. This finding implies that the subtle interplay between oculomotor behavior and neural responses to emotional stimuli is significantly modulated in individuals with SI, providing a refined perspective on their attentional and affective processing deficits. This divergence in rFRP amplitudes under sad conditions further supports the notion that suicide-specific cognitive factors, rather than general negative information processing, are more indicative markers of suicide risk [14].

Moreover, differences in rERP and rFRP under different emotional conditions also highlight how SI uniquely affects the brain’s processing of specific emotions. Surprise, a basic emotion, usually triggers an immediate attention shift to process unexpected stimuli [53]. In the SI group, reduced rERP amplitude in response to surprise after 368 ms might suggest a dampened initial neural response to unexpected emotional cues. This could mean a less effective ability to automatically focus on and integrate new emotional information, impacting how these individuals process social cues or react to unexpected events [51, 54]. Meanwhile, the later-stage blunted response after 450 ms might indicate problems with higher-level cognitive assessment and emotional regulation for surprising situations [55]. Sadness, often linked to prolonged negative emotions, rumination, and altered attention to negative stimuli in depression and SI, typically involves lasting cognitive and emotional engagement [56]. In contrast to the fleeting nature of surprise, sadness requires sustained attention and processing, a condition under which ocular fixation is more pronounced and receptive to rFRP components at 564–920 ms time frame [57, 58].

The differences between rERP and rFRP localized to specific brain regions can be interpreted within the context of known neurocognitive networks involved in emotional processing and attentional control. The SI group showed a lower rERP amplitude at P7 and FT7, which are areas in the temporal lobe (specifically the superior temporal sulcus) known for processing social cues like facial expressions, and the prefrontal cortex, crucial for emotional regulation. This indicates a possibly dampened or altered neural response to unexpected emotional cues during the surprise condition. Besides, the SI group showed a significantly lower rFRP amplitude at electrode PO3, located over the left parieto-occipital cortex. This area is important for visual-spatial attention and processing visual details. Because rFRP is related to microsaccades, changes here could indicate subtle problems with how individuals with SI fine-tune visual input and scan their surroundings, especially when encountering emotional content. This might affect how they focus on or examine negative emotional information.

Overall, the distinct neural responses observed in the SI group, particularly in relation to surprise and sadness, underscore the intricate interplay between emotional processing and suicide risk, suggesting potential biomarkers for early detection and intervention.

### 4.3 The Potential of Multimodal Biomarkers for Suicide Ideation Detection

In the current study, the integration of multimodal data, specifically eye-tracking and EEG measures, was found to enhance the accuracy of detecting SI in individuals with depression compared to relying on eye-tracking measures alone. The improvement in classification performance underscores the complementary nature of these two modalities, each capturing distinct but related aspects of the neurocognitive processes associated with SI. While the EYE model, utilizing only eye-tracking features, achieved an AUC of 0.643, the integration of EEG data in the EYE-EEGmodel resulted in a modest but notable improvement, reaching an AUC of 0.771. This indicates that combining these distinct physiological measures can enhance the accuracy of detecting SI in individuals with depression. Although these AUC values suggest moderate discriminatory ability, they highlight the potential for multimodal approaches in identifying individuals at risk.

Theoretical significance of this multimodal fusion lies in its ability to provide a more comprehensive and nuanced understanding of complex psychiatric conditions like SI, which are inherently multi-faceted and involve interplay across different neurocognitive domains. Eye-tracking provides objective, overt behavioral markers of visual attention and oculomotor control, reflecting how an individual allocates their attention and scans their environment. Conversely, EEG offers a direct window into the underlying neural correlations of these processes, capturing the rapid, subtle changes in brain activity associated with emotional and cognitive processing. Critically, the integration of linear deconvolution modeling into our EEG analysis is a key methodological advancement. Deconvolution allows for the precise disentanglement of overlapping neural signals, such as rERPs from rFRPs. This capability is theoretically significant because it provides cleaner, less confounded measures of specific neural events, enabling a more accurate attribution of brain activity to particular cognitive or emotional processes that might otherwise be obscured by continuous eye movements. By isolating these specific neural components, deconvolution provides theoretically richer EEG features that capture distinct facets of dysfunctional processing in SI.

Therefore, the combined power of eye-tracking and deconvolution-enabled EEG moves beyond merely detecting correlations to building a more robust theoretical model of SI. It allows us to investigate not just what individuals with SI are looking at or how their eyes move, but how their brain is precisely responding to and processing emotional information at a fine-grained neural level in conjunction with these overt behaviors. This holistic approach captures the intricate interplay between behavior and brain activity, providing a richer “biomarker fingerprint” that is likely more reflective of the underlying pathology of SI than any single modality could offer alone. This aligns with the growing recognition in psychiatric research that data-driven multimodal fusion is essential for unraveling the complexity of mental disorders and for developing more effective, biologically-informed diagnostic and prognostic tools [59, 60, 61]. Further research with larger and more diverse samples is needed to refine these models and explore the potential of other biomarkers to improve the accuracy and clinical utility of SI detection.

### 4.4 Limitations

It is important to acknowledge that some of our statistically significant findings, particularly those related to individual eye-tracking measures (e.g., increased fixation on eyes, shorter first fixation duration), exhibited small effect sizes (e.g., η^2^ = 0.004). While these findings contribute to a statistical understanding of attentional biases, their standalone practical or clinical utility as singular biomarkers is limited due to the small proportion of variance they explain. Their primary contribution lies in their role within a more comprehensive, multimodal assessment framework, where they provide complementary information that, when integrated with other measures like EEG, enhances overall predictive accuracy. While our sample size was sufficient to detect differences between groups, larger samples would improve the generalizability of our results and strengthen the predictive models.

A notable limitation of the current study is the significant gender imbalance within our participant sample (89.83% male, 10.17% female). This skew limits the generalizability of our findings, particularly given well-established gender differences in the epidemiology, symptomatology, and neurobiological profiles of depression and SI. For instance, while women are generally more likely to experience depression, men often present with different patterns of suicide behavior and may express SI less directly. Consequently, the neurocognitive markers identified in this predominantly male cohort may not fully translate to female populations, and vice versa. Future research should prioritize recruiting more gender-balanced and diverse samples to validate these findings across genders and explore potential gender-specific neurocognitive markers related to SI.

A limitation is the absence of a healthy NSI group. Our study design enabled a comparison between individuals with depression, with and without suicide thoughts, but it did not allow us to distinguish neurocognitive markers specific to suicide thoughts from those generally present in depression. For instance, some observed changes in emotional processing or attention might be common to all individuals with depression, regardless of suicide thoughts. Future studies should include a healthy NSI group to establish a baseline of normal neurocognitive function and to more accurately identify biomarkers uniquely linked to suicide thoughts in the context of depression. This would improve the specificity and clinical usefulness of any identified risk markers.

Another important consideration is the unaddressed medication status of our participants. Psychotropic medications, particularly antidepressants, are known to significantly influence neurophysiological measures such as EEG activity and oculomotor parameters. While the study did not strictly limit the types or dosages of medication used by patients, the participants in the study were all taking selective serotonin reuptake inhibitors. However, the lack of specific information regarding participants’ medication use, dosage, and duration prevents a definitive determination of the extent to which these factors may have confounded the study’s findings. Future studies should meticulously document and control for medication status, either by recruiting medication-naive participants or by including medication as a covariate in analyses, to ensure that observed differences are attributable to SI rather than pharmacological effects.

Additionally, although we controlled depression heterogeneity, future studies could benefit from dividing participants based on specific symptoms, illness duration, and co-occurring conditions. This would enable the identification of more uniform subgroups and allow for a closer examination of their unique patterns of eye movements and brain activity, potentially offering deeper insights into the neurophysiological factors related to SI and improving the clinical usefulness of the identified biomarkers. Longitudinal studies are also necessary to assess the stability of these biomarkers over time and their ability to predict future suicide behavior.

## 5. Conclusions

In conclusion, this study provides novel evidence for altered neurocognitive processing during face recognition in individuals with depression and SI. By combining eye-tracking, EEG, and deconvolution modeling, we identified distinct patterns of visual attention and emotional processing, as reflected in both behavioral and electrophysiological measures. Specifically, individuals with SI exhibited attentional biases toward emotional faces, coupled with altered neural responses to surprise and sadness. The integration of eye-tracking and EEG data improved the accuracy of detecting SI, highlighting the potential of multimodal biomarkers for identifying individuals at risk. While these findings contribute to our understanding of the neurocognitive mechanisms underlying SI, future research with larger, more diverse samples and longitudinal designs is needed to validate these findings and translate them into clinically useful tools.

## Availability of Data and Materials

The datasets generated and analyzed during the current study, including raw eye-tracking, EEG, and clinical assessment data, are not publicly available due to the sensitive nature of the information and the need to protect participant confidentiality, which would contravene ethical standards and participant privacy agreements. However, de-identified data may be made available from the corresponding author upon reasonable request, subject to ethical review and approval by an institutional review board (IRB) or equivalent ethics committee, and completion of a data access agreement.
